# A genome‐wide association study suggests new evidence for an association of the NADPH Oxidase 4 (*NOX4*) gene with severe diabetic retinopathy in type 2 diabetes

**DOI:** 10.1111/aos.13769

**Published:** 2018-09-04

**Authors:** Weihua Meng, Kaanan P. Shah, Samuela Pollack, Iiro Toppila, Harry L. Hebert, Mark I. McCarthy, Leif Groop, Emma Ahlqvist, Valeriya Lyssenko, Elisabet Agardh, Mark Daniell, Georgia Kaidonis, Jamie E. Craig, Paul Mitchell, Gerald Liew, Annette Kifley, Jie Jin Wang, Mark W. Christiansen, Richard A. Jensen, Alan Penman, Heather A. Hancock, Ching J. Chen, Adolfo Correa, Jane Z. Kuo, Xiaohui Li, Yii‐der I. Chen, Jerome I. Rotter, Ronald Klein, Barbara Klein, Tien Y. Wong, Andrew D. Morris, Alexander S.F. Doney, Helen M. Colhoun, Alkes L. Price, Kathryn P. Burdon, Per‐Henrik Groop, Niina Sandholm, Michael A. Grassi, Lucia Sobrin, Colin N.A. Palmer, Wellcome Trust Case Control Consortium 2 (WTCCC2), Surrogate markers for Micro‐ and Macro‐vascular hard endpoints for Innovative diabetes Tools (SUMMIT) study group

**Affiliations:** ^1^ Division of Population Health Sciences Medical Research Institute Ninewells Hospital and School of Medicine University of Dundee Dundee UK; ^2^ Section of Genetic Medicine Department of Medicine University of Chicago Chicago Illinois USA; ^3^ Department of Epidemiology Harvard T.H. Chan School of Public Health Boston Massachusetts USA; ^4^ Folkhälsan Institute of Genetics Folkhälsan Research Center Helsinki Finland; ^5^ Abdominal Center Nephrology University of Helsinki and Helsinki University Hospital Helsinki Finland; ^6^ Research Programs Unit Diabetes and Obesity University of Helsinki Helsinki Finland; ^7^ Oxford Centre for Diabetes, Endocrinology and Metabolism University of Oxford Churchill Hospital Headington, Oxford UK; ^8^ Wellcome Trust Centre for Human Genetics University of Oxford Oxford UK; ^9^ Oxford NIHR Biomedical Research Centre Churchill Hospital Headington, Oxford UK; ^10^ Department of Clinical Sciences Faculty of Medicine Lund University Malmo Sweden; ^11^ Department of Ophthalmology Royal Melbourne Hospital Parkville Victoria Australia; ^12^ Department of Ophthalmology Flinders University Adelaide South Australia Australia; ^13^ Centre for Vision Research Department of Ophthalmology and Westmead Institute for Medical Research University of Sydney C24 Sydney Australia; ^14^ Cardiovascular Health Research Unit School of Medicine University of Washington Seattle Washington USA; ^15^ Department of Ophthalmology University of Mississippi Medical Center School of Medicine University of Mississippi Jackson Mississippi USA; ^16^ Clinical and Medical Affairs CardioDx Inc. Redwood City California USA; ^17^ Institute for Translational Genomics and Population Sciences Los Angeles Biomedical Research Institute Harbor‐UCLA Medical Center Torrance California USA; ^18^ Department of Pediatrics Harbor‐UCLA Medical Center Torrance California USA; ^19^ Department of Ophthalmology and Visual Sciences University of Wisconsin School of Medicine and Public Health Madison Wisconsin USA; ^20^ Singapore Eye Research Institute Singapore National Eye Centre Yong Loo Lin School of Medicine National University of Singapore Ophthalmology and Visual Sciences Academic Clinical Program Duke‐NUS Medical School Singapore; ^21^ The Usher Institute of Population Health Sciences and Informatics University of Edinburgh Edinburgh UK; ^22^ Medical Research Institute Ninewells Hospital and School of Medicine University of Dundee Dundee UK; ^23^ Institute of Genetics and Molecular Medicine Western General Hospital University of Edinburgh Edinburgh UK; ^24^ Menzies Institute for Medical Research University of Tasmania Hobart Tasmania Australia; ^25^ Department of Diabetes Central Clinical School Monash University Melbourne Victoria Australia; ^26^ Department of Ophthalmology University of Illinois at Chicago Chicago Illinois USA; ^27^ Department of Ophthalmology Massachusetts Eye and Ear Infirmary Harvard Medical School Boston Massachusetts USA; ^28^ Centre for Pharmacogenetics and Pharmacogenomics Medical Research Institute Ninewells Hospital and School of Medicine University of Dundee Dundee UK

**Keywords:** diabetes, diabetic complications, diabetic retinopathy, genome‐wide association study, NOX4

## Abstract

**Purpose:**

Diabetic retinopathy is the most common eye complication in patients with diabetes. The purpose of this study is to identify genetic factors contributing to severe diabetic retinopathy.

**Methods:**

A genome‐wide association approach was applied. In the Genetics of Diabetes Audit and Research in Tayside Scotland (GoDARTS) datasets, cases of severe diabetic retinopathy were defined as type 2 diabetic patients who were ever graded as having severe background retinopathy (Level R3) or proliferative retinopathy (Level R4) in at least one eye according to the Scottish Diabetic Retinopathy Grading Scheme or who were once treated by laser photocoagulation. Controls were diabetic individuals whose longitudinal retinopathy screening records were either normal (Level R0) or only with mild background retinopathy (Level R1) in both eyes. Significant Single Nucleotide Polymorphisms (SNPs) were taken forward for meta‐analysis using multiple Caucasian cohorts.

**Results:**

Five hundred and sixty cases of type 2 diabetes with severe diabetic retinopathy and 4,106 controls were identified in the GoDARTS cohort. We revealed that rs3913535 in the NADPH Oxidase 4 (*NOX4*) gene reached a p value of 4.05 × 10^−9^. Two nearby SNPs, rs10765219 and rs11018670 also showed promising p values (p values = 7.41 × 10^−8^ and 1.23 × 10^−8^, respectively). In the meta‐analysis using multiple Caucasian cohorts (excluding GoDARTS), rs10765219 and rs11018670 showed associations for diabetic retinopathy (p = 0.003 and 0.007, respectively), while the p value of rs3913535 was not significant (p* *=* *0.429).

**Conclusion:**

This genome‐wide association study of severe diabetic retinopathy suggests new evidence for the involvement of the *NOX4* gene.

## Introduction

Diabetic retinopathy (DR) is a chronic, progressive, potentially sight‐threatening disease of the retinal microvasculature associated with pathophysiological changes intensified by diabetes (The Royal College of Ophthalmologists. Diabetic Retinopathy Guidelines 2012). It is the most common eye complication in diabetic patients and the most common cause of blindness among people of working age in the UK (Bunce & Wormald 2008). In general, DR can be broadly graded as non‐proliferative diabetic retinopathy (NPDR) and proliferative diabetic retinopathy (PDR) according to the absence or presence of abnormal new vessels (Williams et al. 2004). In each year, around 60% of diabetic patients under retinal screening have NPDR and approximately 20% of diabetic patients have active or regressed PDR (Keenan et al. 2013). It is estimated that over 1000 new cases of blindness are caused by DR each year in England alone and a further 4000 people each year in the country are thought to be at risk of vision loss due to retinopathy (http://www.diabetes.co.uk 2017). Around one in five type 2 diabetic patients in Scotland have been diagnosed with DR, and 10% of these DR patients need to be referred to ophthalmologists for further treatment (Looker et al. 2012). The quality of life can be significantly affected for DR patients due to visual impairment, worries and movement restrictions (Woodcock et al. 2004). In addition to physical and emotional impacts, DR also represents a significant economic burden to the healthcare system. In the USA, the total direct medical cost of DR in adults was estimated to be $493 million per year (Rein et al. 2006). The average annual healthcare costs of NPDR and PDR per person were €26 and €257 in Sweden, respectively (Heintz et al. 2010). In addition to the fact that there is a huge cost difference depending on the severity of DR, It has been estimated that without treatment for PDR, 50% of all patients will become blind within 5 years following diagnosis (Williams et al. 2004). Therefore, it is essential to identify and treat this disorder at an early stage and slow or stop its progress.

Epidemiological studies have proposed multiple risk factors associated with the development and the progression of DR from longitudinal studies including higher glycaemia, higher blood pressure, no smoking, male sex, higher HbA1c, longer duration of diabetes, lower body mass index and elevated blood urea concentration (Stratton et al. 2001; Xu et al. 2012). In addition, Xu et al. (2012) reported that patients with microalbuminuria were 4.7 times more likely to have a severe or proliferating DR than those without microalbuminuria. This study links DR with renal function since both DR and diabetic nephropathy are microvascular complications of diabetes. As most of these risk factors are amenable, clinical trials have been performed to intervene with the incidence and progress of DR. It is reported that intensive blood sugar control effectively delays the onset and slows the progression of DR when initiated in adolescent subjects (Diabetes & Complications 1994; White et al. 2001). The persistent beneficial effect in DR in the intensive therapy group continues for at least 10 years (White et al. 2008). Epidemiological studies are one of the essential tools to identify risk factors and effective preventive strategies for diseases along with genetic studies which search for underlying causative biological mechanisms and genetic pathways.

Understanding the genetic factors associated with DR would assist in identifying biological underpinnings and potentially suggest molecular targets for pharmacological research. Both twin studies and family studies have confirmed that DR is a heritable trait in both type 1 and type 2 diabetes (Leslie & Pyke 1982; Rema et al. 2002; Hallman et al. 2005; Looker et al. 2007; Monti et al. 2007; Zhang et al. 2010). In particular, a greater genetic component exists in more severe types of DR (Hallman et al. 2005). The heritability of DR has been estimated to be 18% in sibling samples (Looker et al. 2007). Genome‐wide linkage studies have proposed multiple chromosome loci to be linked with DR using multiple ethnic groups, while no specific genes have been identified (Imperatore et al. 1998; Hallman et al. 2007; Looker et al. 2007). Candidate gene approaches have suggested promising genes with possible biological connections with DR such as *VEGFA*,* AKR1B1*,* AGER*,* ICAM1*,* MTHFR*, while larger samples will be required to further consolidate the reliability of these results (Awata et al. 2002; Lindholm et al. 2006; Abhary et al. 2010; Simões et al. 2014; Opatrilova et al. 2017).

Genome‐wide association studies (GWAS) have been very successful in identifying potential candidate genes for common complex disorders using DNA chips (McCarthy et al. 2008). Recently, several GWAS have proposed multiple susceptibility loci for DR. However, none of these loci achieved genome‐wide significance, and none have been replicated by other studies (Fu et al. 2010; Grassi et al. 2011; Huang et al. 2011; Sheu et al. 2013; Burdon et al. 2015).

To facilitate identification of the genetic factors associated with severe DR, we performed this GWAS using a homogenous Scottish diabetic population in the first stage and multiple Caucasian DR cohorts in the replication stage. To our knowledge, this is the first GWAS based on Scottish Diabetic Retinopathy Grading Scheme and the largest GWAS on severe DR so far.

## Patients and Methods

### Participants In the discovery cohort

The datasets from the GoDARTS project were analysed in this study. The GoDARTS project mainly recruits type 2 diabetic patients and non‐diabetic controls throughout Tayside, Scotland, to identify genetic susceptibility to diabetes including its complications and response to treatment. Participants will undertake a simple baseline clinical examination and complete a lifestyle questionnaire in addition to providing biological samples such as blood and urine. The participants provide informed consent at the time of recruitment which allows the use of their data and samples (including extracted DNA) for research purposes as well as link the data anonymously to their medical records. These records include the Scottish Care Information‐Diabetes Collaboration (SCI‐DC) and Scottish Diabetic Retinopathy Screening Collaborative electronic health records used by health care professionals throughout Scotland for the care of patients with diabetes. Further information, including data access procedures, can be found at http://diabetesgenetics.dundee.ac.uk/. The GoDARTS study has been approved by Tayside Committee on Medical Research Ethics, and informed consent was obtained from all patients (REC reference 053/04). The research adhered to the tenets of the Declaration of Helsinki.

So far, the project has recruited 9,439 diabetic patients and 6,927 of them have been genotyped. For this study, we extracted the DR screening records of all GoDARTS individuals from June 1996 until June 2011 as well as information on age, gender, body mass index (BMI), HbA1c and duration of diabetes.

### DR grading in Scotland

Retinal screening has been undertaken in Tayside since 1990, and the DR screening protocol has previously been described (Leese et al. 2005). According to the Scottish Diabetic Retinopathy Grading Scheme, DR status can be graded into five levels: level R0: No DR; level R1: mild background retinopathy; level R2: moderate background retinopathy; level R3: severe background retinopathy; level R4: PDR. The detailed diagnostic criteria are summarized in Liu et al. (2013)'s paper. In addition, the status of macula was recorded as with or without diabetic maculopathy. However, the status of the macula was not taken into account in this study when defining DR. The history of laser photocoagulation treatment was also recorded for GoDARTS participants but lacking information of the exact methods (panretinal photocoagulation, focal photocoagulation or both).

There are other DR grading systems such as Early Treatment Diabetic Retinopathy Study (ETDRS), American Academy of Ophthalmology (AAO) and National Screening Committee (NSC). The approximate equivalence of Scottish Diabetic Retinopathy Grading Scheme and alternative classification systems for diabetic retinopathy can be found in the latest DR guideline from The Royal College of Ophthalmologists (The Royal College of Ophthalmologists 2012).

### Definition of severe diabetic retinopathy cases and controls in GoDARTS

A severe DR case was defined in this study as a type 2 diabetic individual with at least one eye that has previously been coded as severe background retinopathy (level R3) or PDR (level R4); or with a history of laser photocoagulation treatment in the e‐health records.

A control was defined as a type 2 diabetic individual with DR longitudinal screening records for both eyes, which were only graded as normal (level R0) or mild background retinopathy (level R1). In addition, controls had no record of laser photocoagulation treatment.

To maintain homogeneous case and control populations, we removed type 2 diabetic individuals whose severest DR screening records were moderate background retinopathy (level R2) from both cases and controls.

In simple words, this study compared severe DR cases (level R3 and R4) with controls (level R0 and R1).

### DR definitions in the multiple Caucasian and African American Cohorts

Meta‐analyses were performed in four studies of Caucasian patients with type 2 diabetes (The Scania Diabetes Registry, The Australian DR Genetics Case‐Control Study, The Blue Mountain Eye Study and Cardiovascular Health Study 2) and two studies of Caucasian patients with type 1 diabetes (The Finnish Diabetic Nephropathy Study and The Genetics of Kidneys in Diabetes study/The Epidemiology of Diabetes Interventions and Complications). Diabetic retinopathy (DR) was defined either based on ETDRS scoring, or laser treatment. See the Appendix S1 for the DR definitions for all cohorts. A general description of all cohorts can also be found in the Appendix S1.

### Genotyping and quality control

The GoDARTS diabetic individuals were genotyped by either Affymetrix SNP6.0 chips (3,673 patients) funded by the Wellcome Trust Case Control Consortium 2 (WTCCC2) project or by Illumina OmniExpress chips (3,254 patients) funded by the Surrogate markers for Micro‐ and Macro‐vascular hard endpoints for Innovative diabetes Tools (SUMMIT) project. Standard protocols were used for genotyping quality controls for the WTCCC2 studies and the SUMMIT studies (GoDARTS and UKPDS Diabetes Pharmacogenetics Study Group et al. 2011; Fagerholm et al. 2012). The genotyping quality control of other Caucasian and African American DR cohorts followed their own protocols.

### Statistical analysis

The imputation of non‐genotyped single nucleotide polymorphisms (SNPs) in the Affymetrix SNP6.0 chips and Illumina OmniExpress chips were done by SHAPEIT and IMPUTE2 using reference files from the 1000 genome phase I datasets (Howie et al. 2006; Delaneau et al. 2011). The recommended *r* ^2^ > 0.3 was used to filter out badly imputed SNPs. Routine quality control steps were frequently applied using PLINK (removing SNPs with over 5% genotyping missing, or with minor allele frequency less than 1%, or those that failed Hardy–Weinberg tests p* *<* *0.000001, and removing individuals with more than 5% genotype data missing) (Purcell et al. 2007). SNPs on the X and Y chromosomes and mitochondrial SNPs were excluded from analyses. Population stratification analysis was based on multidimensional scaling integrated in PLINK to detect any difference in ancestry within the cohort, with a lambda value indicating the level of stratification. Removal of related samples was based on pi‐hat > 0.125 in PLINK. The p values for SNP associations were generated based on logistic regression analyses using PLINK, and adjusting for age, gender, BMI,HbA1c and duration of diabetes. A p value of less than 5 × 10^−8^ was considered to be an association, warranting further exploration. The LD scores (*R*‐squared) among significant SNPs were later calculated by PLINK. The positive SNPs generated from the first stage were then meta‐analysed using multiple replication cohorts where p values of these SNPs were generated by logistic regression adjusting with relevant covariates. Multiple meta‐analyses were performed by GWAMA combining Caucasian cohorts and African American cohorts (Mägi & Morris 2010). SNP functional annotations were applied by SNPnexus, and the Manhattan plot was generated by HaploView (Barrett et al. 2005; Dayem Ullah et al. 2013). LocusZoom was used for regional visualization (Pruim et al. 2010). SNPEVG was used to generate the corresponding Q‐Q plot, a tool to evaluate differences between cases and controls caused by potential confounders (different genotyping lab, different DNA extraction methods, etc) (Wang et al. 2012). Narrow‐sense heritability was calculated by GCTA (Lee et al. 2011). Means of age, BMI, HbA1c and duration of diabetes were compared between cases and controls using independent t tests in spss 22 (IBM Corp, New York, NY, USA). The gender difference was evaluated using Chi‐squared test (2 × 2 tables).

## Results

**Figure 1:**
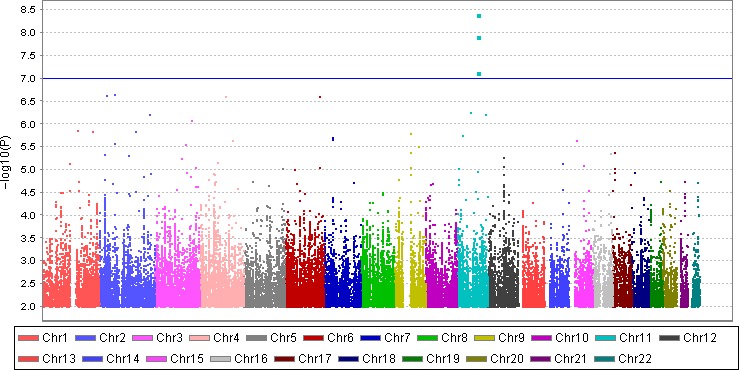
Manhattan plot of the GWAS on severe DR using quality‐controlled SNPs. *X* axis represents 22 autosomes. *Y* axis means the −log10 of p values. The blue line indicates the p value of 10^−7^.

**Figure 2:**
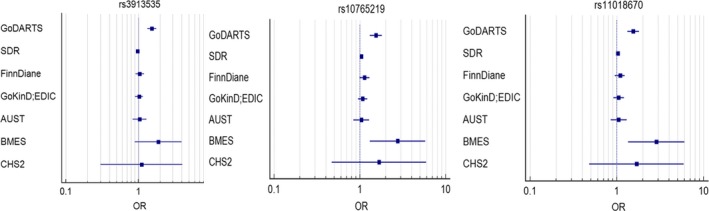
The forest plots of 3 SNPs in the Caucasian DR cohorts.

In the GoDARTS population, we identified 560 unrelated severe DR patients (133 individuals with severe background retinopathy and 427 individuals with PDR) and 4106 controls (1873 individuals with no DR and 2233 individuals with mild background retinopathy) based on our definitions. The clinical characteristics of cases and controls are summarized in Table S1. There are statistical differences between the cases and the controls in terms of gender, age, duration of diabetes and HbA1c. There is no statistical difference of BMI between the two groups. Altogether 6 585 471 imputed SNPs passed from routine quality control checking and imputation quality score *r* ^2^ > 0.3. Since the multidimensional scaling analysis for population stratification found a lambda value of 1.005 for the cleaned datasets, no further adjustment based on population stratification was applied. The corresponding Q‐Q plot is shown in Fig. S1. Using logistic regression analysis integrated in PLINK with gender, age, duration of diabetes and HbA1c as covariates, there was a cluster appearing in the Manhattan plot (only SNPs with p values less than 0.01 were used) (Fig. 1). The top SNP in this region was rs3913535 in the *NOX4* gene with a p value of 4.05 × 10^−9^ and an odds ratio (OR) of 1.55 (95% confidence interval: 1.34–1.79). Two nearby SNPs, rs10765219 and rs11018670, also showed promising p values (p* *= 7.41 × 10^−8^, 1.23 × 10^−8^, respectively). Table 1 summarises these SNPs found in the region. Figure S2 shows the regional plot of the identified loci. We downloaded the linkage information of these three SNPs from HapMap Caucasian population and found the linkage disequilibrium (LD) scores (*R*‐squared) of these three SNPs from GoDARTS are quite consistent with those from HapMap (Table S2). The heritability of severe DR was estimated to be 7.00% in this diabetic population based on the restricted maximum likelihood analysis by GCTA. In the replication cohorts, including seven Caucasian DR cohorts with multiple DR definitions, the meta‐analysis p values of rs3913535, rs10765219 and rs11018670 were 0.429, 0.003 and 0.007, respectively (Table 2). When combined with the GoDARTS results, the meta‐analysis p values of these three SNPs were 0.71, 9.02 × 10^−5^ and 4.24 × 10^−4^, respectively (Table 2). The forest plots of these three SNPs in the Caucasian DR cohorts are summarized in Fig. 2. In the four African American DR cohorts, the meta‐analysis p values of rs3913535, rs10765219 and rs11018670 were 0.883, 0.814 and 0.686, respectively (Table S3).

**Table 1:** Top hits of the GWAS on the severe DR in the GoDARTS

SNPs	Chr:position (hg19)	Gene	Minor allele (its frequency in cases:controls)	p value	OR ± SE	CI 0.95	Imputation score (Affymetrix:Illumina)
rs3913535	11:89096757	*NOX4*	C (0.518:0.411)	4.05 × 10^−9^	1.55 ± 0.07	1.34–1.79	1:0.98
rs10765219	11:89354278	31 kb to *NOX4*	T (0.529:0.424)	7.41 × 10^−8^	1.54 ± 0.08	1.31–1.80	1:0.94
rs11018670	11:89356628	33 kb to *NOX4*	G (0.534:0.427)	1.23 × 10^−8^	1.55 ± 0.08	1.33–1.80	1:0.98

CI = confidence interval, OR ± SE = odds ratio ± standard error.

**Table 2:** The input information and the output meta‐analysis results of 7 Caucasian cohorts based on GWAMA (default setting)

Cohorts	MARKER	EA	NEA	OR	OR_95L	OR_95U	*N* (cases + controls)	p
GoDARTS (severe DR, type 2)	rs3913535	C	T	1.55	1.34	1.79	4666 (560 + 4,106)	4.05 × 10^−9^
	rs10765219	T	G	1.54	1.31	1.80	4666	7.41 × 10^−8^
	rs11018670	G	A	1.55	1.33	1.80	4666	1.23 × 10^−8^
SDR (general DR, type 2)	rs3913535	C	T	0.98	0.95	1.01	2016 (1,151 + 865)	0.25
	rs10765219	T	G	1.04	1.004	1.07	2016	0.02
	rs11018670	G	A	1.03	1.004	1.07	2016	0.03
AUST (general DR, type 2)	rs3913535	C	T	1.05	0.84	1.30	780 (346 + 434)	0.68
	rs10765219	T	G	1.04	0.84	1.28	780	0.73
	rs11018670	G	A	1.05	0.85	1.30	780	0.62
BMES (general DR, type 2)	rs3913535	C	T	1.89	0.90	3.99	162 (15 + 147)	0.09
	rs10765219	T	G	2.76	1.31	5.79	162	0.007
	rs11018670	G	A	2.85	1.35	6.02	162	0.006
CHS2 (general DR, type 2)	rs3913535	C	T	1.12	0.30	4.11	116 (5 + 111)	0.87
	rs10765219	T	G	1.67	0.47	5.92	116	0.43
	rs11018670	G	A	1.69	0.48	5.93	116	0.42
FinnDiane (general DR, type 1)	rs3913535	C	T	1.05	0.92	1.21	2670 (1,638 + 1,032)	0.53
	rs10765219	T	G	1.13	0.99	1.29	2670	0.08
	rs11018670	G	A	1.09	0.95	1.23	2670	0.23
GoKinD; EDIC (general DR type 1)	rs3913535	C	T	1.03	0.91	1.16	2829 (973 + 1,856)	0.52
	rs10765219	T	G	1.08	0.95	1.22	2829	0.21
	rs11018670	G	A	1.05	0.91	1.21	2829	0.42
Meta‐analysis without GoDARTS	rs3913535	C	T	0.99	0.96	1.02	8573 (4,128 + 4,445)	0.42
	rs10765219	T	G	1.05	1.02	1.08	8573	0.003
	rs11018670	G	A	1.03	1.01	1.06	8573	0.007
Meta‐analysis all Caucasian cohorts	rs3913535	C	T	1.01	0.98	1.03	13 239 (4688 + 8,551)	0.71
	rs10765219	T	G	1.07	1.03	1.10	13 239	9.02 × 10^−5^
	rs11018670	G	A	1.04	1.02	1.07	13 239	4.24 × 10^−4^

AUST = The Australian DR Genetics Case–Control Study, BMES = The Blue Mountain Eye Study, CHS2 = Cardiovascular Health Study 2, EA = effective allele, EDIC = The Epidemiology of Diabetes Interventions and Complications, FinnDiane = The Finnish Diabetic Nephropathy Study, GoDARTS = The Genetics of Diabetes Audit and Research Tayside, GoKinD = The Genetics of Kidneys in Diabetes study, *N *= number, NEA = non‐effective allele, OR = odds ratio, SDR = The Scania Diabetes Registry.

## Discussion

This GWAS on severe DR was based on a well‐defined type 2 diabetic population in the UK and has suggested new evidence that the *NOX4* gene might be associated with severe DR. Supporting evidence was also obtained from multiple Caucasian DR cohorts.

In Scotland, all patients with diabetes are invited to have an annual retinal screening. During the screening, the clinical information of the eyes is recorded such as visual acuity (VA), cataract status, retinal status, macula status, etc. The severe DR cases in GoDARTS are a combination of type 2 diabetes patients with severe background retinopathy and PDR or anyone with a record of laser photocoagulation treatment. We have two reasons for this definition. First, Liu et al. (2013) observed that there is an accelerated trend from a less severe DR status to a more severe DR status. For example, it takes approximately 0.11 years for severe background retinopathy to progress to PDR, while it takes 12.6 years from no DR status to mild background retinopathy. In other words, most severe background retinopathy will progress to PDR in a relatively short timeframe. This is matched with the fact that there were more PDR (427 individuals) than severe background retinopathy (133 individuals) in the GoDARTS cohort. Second, Grassi et al. used patients with PDR and/or diabetic macular oedema as severe DR cases. Though this case definition is stricter, it only left 281 severe cases for their sub‐GWAS analysis on severe DR (Grassi et al. 2011). Therefore, it is reasonable to use our definition to increase case sample size and correspondingly to increase the power of this study. Our control definition includes DR‐free and mild background retinopathy individuals. This is based on a phenomenon that substantial rates of mild background DR regression can be observed in longitudinal studies. Diabetic retinopathy (DR) is often observed to regress from mild background retinopathy to no DR status, but it is not possible to revert from PDR to no DR (Liu et al. 2013). Around 50% of type 2 diabetic patients without DR at baseline will develop DR five years later, while 25% of type 2 diabetic patients with non‐severe DR will fully recover from DR in the same period (Jin et al. 2014). Since these patients fluctuate between DR‐free and mild background retinopathy, it is reasonable to treat them as one group. Further, the genetic mechanisms of non‐severe DR and severe DR might not be the same. Non‐severe DR is likely driven by a microenvironment change in the eye, while severe DR can be the consequences of the interaction between genes and the microenvironment in the eye. In other words, genetics play a bigger role in severe DR than non‐severe DR (Hallman et al. 2005). The same phenomenon also happens in another eye disorder—myopia, for which low myopia is considered as an environmental‐driven result, while high myopia is a consequence of both genetic and environment factors (Meng et al. 2012a, b). Thus, it is reasonable to use this control definition. Nevertheless, our control population with no severe DR cases is better than using a population control which contains a small proportion of severe DR cases. To have homogeneous case and control populations, we also removed moderate background retinopathy samples from both case and control populations. In the GWAS study on type 1 diabetes by Grassi et al., all non‐severe DR cases were treated as control samples, which means they support our hypothesis that genetic mechanisms of severe DR and non‐severe DR might not be the same. Similar to Grassi et al.'s work, we also do not consider the status of diabetic nephropathy in our GWAS. We recognize that our case‐control ratio is 1:7 which is consistent with the case‐control ratio of the UKPDS cohort (R0 = 2,316, R1 + R2 = 801, R3 + R4 = 509) (Kohner et al. 2001). It is expected that the power will not increase dramatically if the case‐control ratio is smaller than 1:4 when the overall sample size increases (Grimes & Schulz 2005). But, for GWAS, it is recommended to have a larger sample size. A good phenotype definition will gather relatively homogeneous individuals with similar clinical conditions and underlying genetic mechanisms.

The most significant SNP was identified in the *NOX4* gene with a p value of 4.05 × 10^−9^ at rs3913535 and an odds ratio (OR) of 1.55 (95% confidence interval: 1.34–1.79). Two nearby SNPs, located next to *NOX4*, rs10765219 and rs11018670 also showed promising p values (p* *= 7.41 × 10^−8^, 1.23 × 10^−8^). The *NOX4* gene encodes the NOX4 protein which is located in non‐phagocytic cells where it acts as an oxygen sensor and catalyses the reduction of molecular oxygen to various reactive oxygen species (ROS). The ROS has been linked with numerous biological functions including signal transduction, cell differentiation and tumour cell growth (http://www.ncbi.nlm.nih.gov/gene/50507 2017). Nox4 mediates vascular endothelial growth factor receptor (VEGF) 2‐induced intravitreal neovascularization in a rat model of retinopathy of prematurity (Wang et al. 2014). In mice models, activation of Nox4 plays an essential role in high‐glucose and hypoxia‐mediated VEGF expression and diabetes‐induced blood‐retinal barrier breakdown, while the inhibition of Nox4 contributes to the protective effects of lovastatin in diabetic retinopathy (Li et al. 2010). In addition, Nox4's expression is significantly increased in oxygen‐induced retinopathy and upregulation of Nox4 contributes to retinal neovascularization formation in oxygen‐induced retinopathy (Li et al. 2014). Further, Nox4 has been identified to mediate insulin‐stimulated VEGF expression and angiogenesis in cells (Meng et al. 2012a). Last but not least, it is also reported that Nox4‐mediated oxidative stress contributes to Wnt pathway activation in diabetic retinopathy (Liu et al. 2014). Modulation of retinal Nox4 expression may present a promising therapeutic approach for neovascular retinal diseases. In addition to DR, *NOX4* has been linked with other diabetic microvascular disorders such as diabetic nephropathy with strong evidence. Inhibitor of the *NOX4* gene has been proved to have renoprotection in diabetic nephropathy (Gorin & Block 2013; Jha et al. 2014; Thallas‐Bonke et al. 2014). The inhibitor, also named as GKT‐137831, has been under Phase II clinical development for the treatment of diabetic nephropathy, though it failed to pass recently (Gorin et al. 2015). We did not have direct replications of these 3 SNPs for the same definitions of severe DR, but we have performed a meta‐analysis of these 3 SNPs from multiple Caucasian DR cohorts including Scania Diabetic Registry (SDR), Finnish Diabetic Nephropathy Study (FinnDiane), Genetics of Kidneys in Diabetes study (GoKinD), Epidemiology of Diabetes Interventions and Complications (EDIC), Australian DR Genetics Case‐Control Study (AUST), The Blue Mountain Eye Study (BMES) and Cardiovascular Health Study 2 (CHS2), regardless of the types of diabetes, the DR grading methods and the severity of DR (Table 2). The meta‐analysis p values of these SNPs were no longer GWAS significant though the p values of rs10765219 and rs11018670 were less than 0.01. This could be due to multiple reasons such as different ethnic populations, different types of diabetes, the heterogeneity of DR definitions, different levels of DR severity in these Caucasian DR cohorts and different adjusted covariates (which might lead to bias) among cohorts (see the Appendix S1). We also performed a meta‐analysis using four African American DR cohorts including African American Proliferative Diabetic Retinopathy Study (AAPDR), Jackson Heart Study (JHS), Atherosclerosis Risk in Communities (ARIC) Study and Multi‐Ethnic Study of Atherosclerosis‐African Americans (MESA‐AA). The three SNPs did not show positive results (Table S3). The directions of the effect of these SNPs are quite consistent among Caucasian DR cohorts, while they were quite mixed among African American DR cohorts. In addition to the above‐mentioned reasons, the smaller sample size in each African American cohort could be causing these mixed directions of effect. Finally, it is noticed that all the three SNPs strongly affect the *NOX4* gene expression according to the Genotype‐Tissue Expression (GTEx) portal though the cells used were from fibroblasts, not from eyes (Carithers et al. 2015).

Narrow‐sense heritability of severe DR was estimated to be 7.00% in this diabetic population. Narrow‐sense heritability means the ratio of total phenotypic variance that is due to additive genetic effects (Lee et al. 2011). This estimation does not include the contribution of gene‐gene interactions, gene‐environment interactions, etc, so the actual heritability of this phenotype is likely to be greater. We have suggested that severe DR is a heritable trait in this GWAS and further genetic research is warranted.

We had moderate power in this study due to the limited number of cases in GoDARTS. According to CaTS, using an additive model, we had 80% power to detect a genotypic relative risk of 1.50 for variants with a minor allele frequency of 30% when the disease prevalence in the population is 25% and the significance level is 5 × 10^−8^ (Skol et al. 2006). We also provided the corresponding p values in the GoDARTS of the SNPs suggested by other GWAS studies (Table S4). For reader's interest, we also selected controls with diabetic history over 20 years (*N *=* *470) to perfectly match cases in terms of duration of diabetes. The p values of the three SNPs increased, which were mainly caused by decreased sample size.

A consensus phenotyping approach to DR will not only improve data and study quality but also help to discover novel mechanisms of DR at a molecular level. It has the potential for identifying drug targets and eventually leading to better therapeutic management. This study will initiate more questions to answer about diabetic retinopathy, such as 1. Do diabetic retinopathy and other types of retinopathies share common genetic components? 2. Are the mechanisms of diabetic retinopathy in type 1 and type 2 diabetes the same or not?

This analysis suggests new evidence that *NOX4* gene might be associated with severe DR. We used a novel approach in this study to define severe DR cases and controls, based on DR screening results and Scottish Diabetic Retinopathy Grading Scheme to have a reasonably homogenous phenotype. Our next step is to attempt replication of significant SNPs using newly recruited samples and focus on the molecular mechanisms that may be responsible for the association hits. The findings of these studies will help to confirm the role of *NOX4* in the DR mechanisms and provide possible drug targets for DR treatment.

## Supporting information


**Figure S1.** Q‐Q plot expected and observed log10(1/*P)* values.

**Figure S2.** Regional plot of NOX4 gene area.

**Appendix S1.** DR definitions in the multiple Caucasian and African American DR cohorts.

**Table S1.** The clinical characteristics of the case and control populations in GoDARTS.

**Table S2.** Linkage disequilibrium (LD) score in the GoDARTS and in the HapMap CEU populations.

**Table S3.** The input information and the output meta‐analysis results of 4 African American DR cohorts based on GWAMA (default setting).

**Table S4.** The p values of suggestive SNPs (identified by others) in GoDARTS.

**Table S5.** The p values of the 3 SNPs when controls (with diabetic history over 20 years) were matched with cases in terms of duration of diabetes.
