# Supplementary material for: A genome‐wide association study suggests new evidence for an association of the NADPH Oxidase 4 (NOX4) gene with severe diabetic retinopathy in type 2 diabetes
Source: Acta Ophthalmol. 2018 Sep 4;96(7):e811–9. doi: 10.1111/aos.13769 (PMC6263819; doi:10.1111/aos.13769)
Supplement: Supplementary file 1 — Figure S1. Q‐Q plot expected and observed log10(1/P) values. [file AOS-96-e811-s001.pptx]

## Slide 1
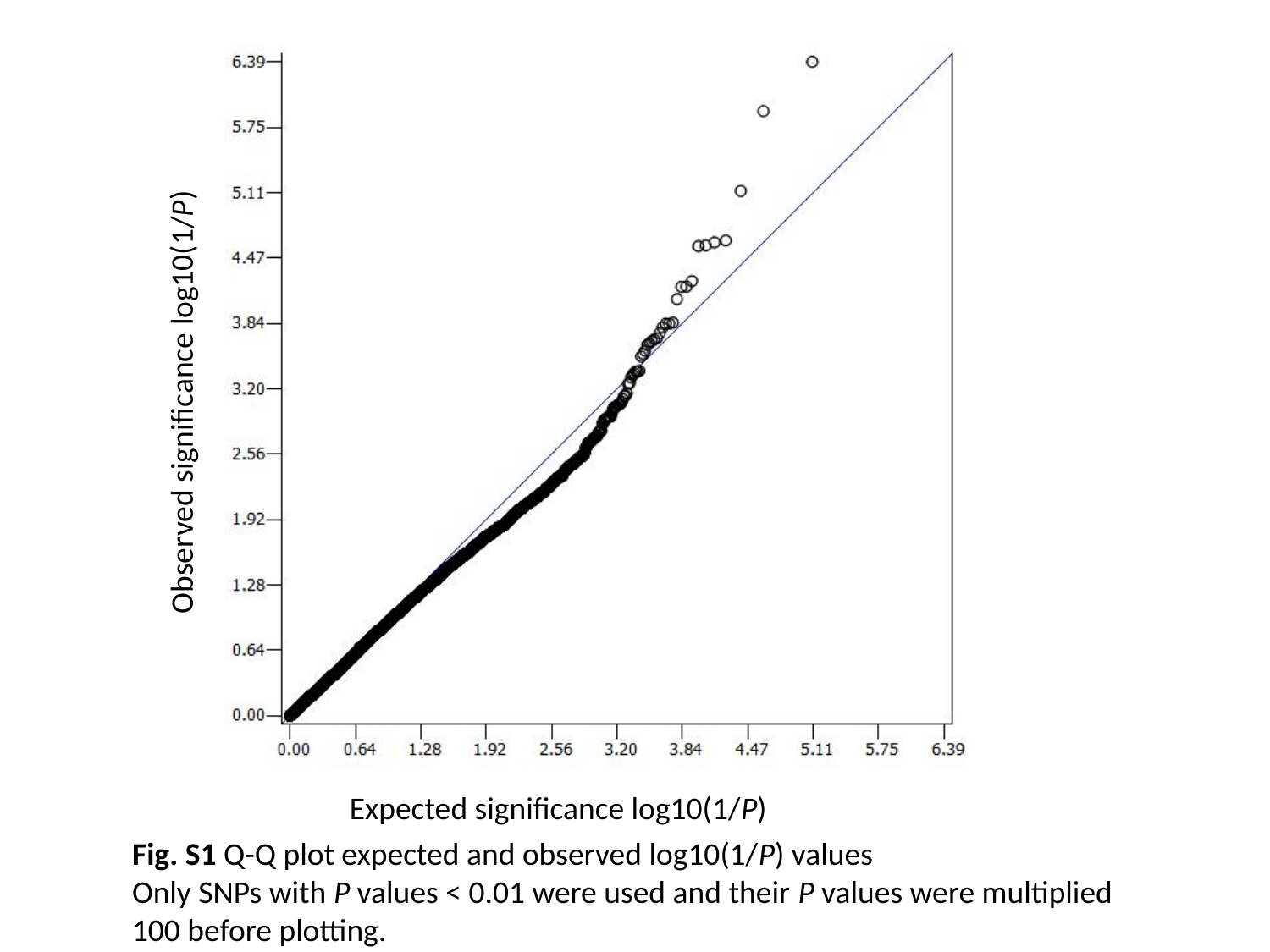

Observed significance log10(1/P)
Expected significance log10(1/P)
Fig. S1 Q-Q plot expected and observed log10(1/P) values
Only SNPs with P values < 0.01 were used and their P values were multiplied 100 before plotting.
