# Supplementary material for: A genome‐wide association study suggests new evidence for an association of the NADPH Oxidase 4 (NOX4) gene with severe diabetic retinopathy in type 2 diabetes
Source: Acta Ophthalmol. 2018 Sep 4;96(7):e811–9. doi: 10.1111/aos.13769 (PMC6263819; doi:10.1111/aos.13769)
Supplement: Supplementary file 2 — Figure S2. Regional plot of NOX4 gene area. [file AOS-96-e811-s002.pptx]

## Slide 1
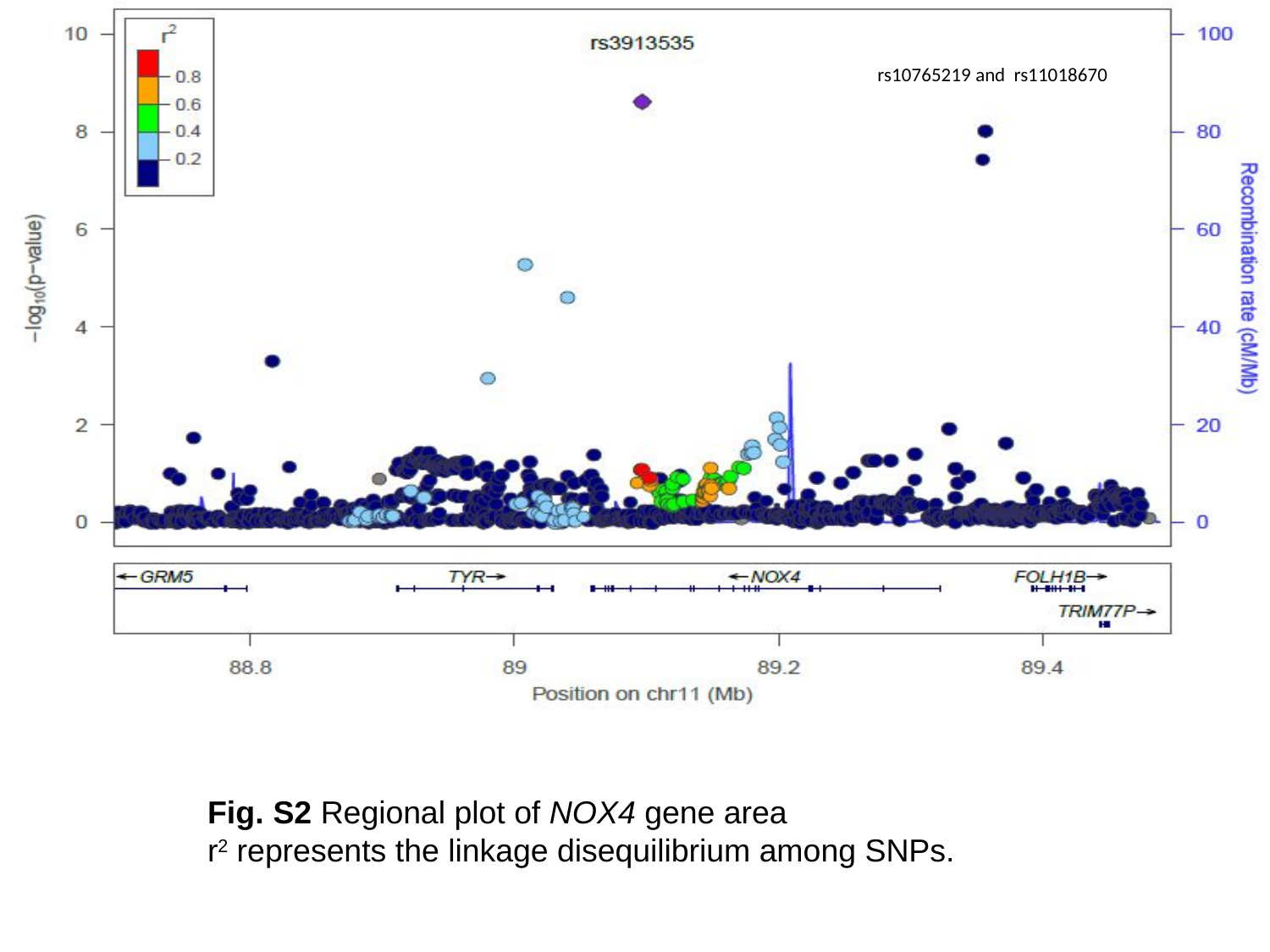

rs10765219 and rs11018670
Fig. S2 Regional plot of NOX4 gene area
r2 represents the linkage disequilibrium among SNPs.
