# Supplementary material for: A genome‐wide association study suggests new evidence for an association of the NADPH Oxidase 4 (NOX4) gene with severe diabetic retinopathy in type 2 diabetes
Source: Acta Ophthalmol. 2018 Sep 4;96(7):e811–9. doi: 10.1111/aos.13769 (PMC6263819; doi:10.1111/aos.13769)
Supplement: Supplementary file 3 — Appendix S1. DR definitions in the multiple Caucasian and African American DR cohorts. [file AOS-96-e811-s003.docx]

**DR definitions in the multiple Caucasian and African American DR cohorts**

**DR Definitions for SDR：**

DR cases: type 2 diabetic patients with panretinal laser therapy, or at least moderate background retinopathy, or mild background retinopathy with duration of diabetes at retinopathy assessment <10 years.

Controls: patients with no recorded background retinopathy, maculopathy or panretinal laser therapy. These individuals should have at least 4 years duration of diabetes.

**DR definitions for FinnDiane:**

Cohort inclusion criteria for both cases and controls: adult participant (age > 18 years) with type 1 diabetes, age at diabetes onset > 40.DR

Cases: type 1 diabetic patients with panretinal laser therapy.

Controls: patients with no records of panretinal laser treatment, and diabetes duration > 20 years.

1. Sandholm N, Van Zuydam N, Ahlqvist E et al. The Genetic Landscape of Renal Complications in Type 1 Diabetes. J Am Soc Nephrol. 2017;28:557-574.

**DR definitions for GoKinD and EDIC:**

DR definitions: severe type 1 diabetic retinopathy as defined by diabetic macular edema or proliferative diabetic retinopathy in unrelated cases. In GoKinD this determination was made by self-report and in EDIC it was by: 'photographic and clinical evidence of prior laser treatment for diabetic eye disease including focal laser treatment and panretinal photocoagulation’.

Controls: all the remaining type 1 diabetic subjects in the cohorts.

The definitions were based on the following two publications.

1. Grassi MA, Sun W, Gangaputra S et al. Validity of self-report in type 1 diabetic subjects for laser treatment of retinopathy. Ophthalmology. 2013;120:2580-2586.
2. Grassi MA, Mazzulla DA, Knudtson MD et al. Patient self-report of prior laser treatment reliably indicates presence of severe diabetic retinopathy. Am J Ophthalmol. 2009;147:501-504.

**DR definitions for BMES, CHS2, AAPDR, JHS, ARIC, MESA-AA:**

Only patients with documented durations of diabetes were included. For these patients:

Cases were defined as ETDRS >= 40, regardless of diabetes duration or ETDRS >=20 with diabetes duration of <= 10 years.

Controls were defined as having an ETDRS <=10 and duration of diabetes >=4 years

**DR definitions for AUST**

Patients of at least 18 years of age who were receiving medical treatment for type 2 diabetes mellitus for at least 5 years were invited to participate. Retinopathy status was determined from direct ophthalmic examination by the treating ophthalmologist and was graded according to modified ETDRS criteria.

Only patients with documented durations of diabetes were included. For these patients:

Cases were defined as >= moderate NPDR, regardless of diabetes duration or mild NPDR with diabetes duration of <= 10 years.

Controls were defined as having no DR and duration of diabetes >=5 years

1. Kaidonis G, Abhary S, Daniell M et al. Genetic study of diabetic retinopathy: recruitment methodology and analysis of baseline characteristics. Clin Exp Ophthalmol. 2014;42:486-493.

**General descriptions of all participating cohorts**

**GoDARTS** (The Genetics of Diabetes Audit and Research Tayside): The GoDARTS project mainly recruits type 2 diabetic patients and non-diabetic controls throughout Tayside, Scotland to identify genetic susceptibility to diabetes including its complications and response to treatment. Participants will undertake a simple baseline clinical examination and complete a lifestyle questionnaire in addition to providing biological samples such as blood and urine. The participants provide informed consent at the time of recruitment which allows the use of their data and samples (including extracted DNA) for research purposes as well as link the data anonymously to their medical records. These records include the Scottish Care Information-Diabetes Collaboration (SCI-DC) and Scottish Diabetic Retinopathy Screening Collaborative – electronic health records used by health care professionals throughout Scotland for the care of patients with diabetes. Further information, including data access procedures, can be found at <http://diabetesgenetics.dundee.ac.uk/>.

**SDR** (The Scandia Diabetes Registry): The Scania Diabetes Registry contains over 7000 diabetes patients recruited at hospitals in Scania, Sweden as from 1996. The majority of the patients come from the city of Malmö, and they account for about 25% of all diabetic patients in the region. At the clinic, all patients with Diabetes Mellitus were asked if they would like to participate in Scania Diabetes Registry. The diagnosis of the diabetes was made in the clinic, and the majority of patients were on antidiabetic treatment. Additional information has been added from the routinely clinical follow-ups. Information about onset of diabetes, and mode of treatment, BMI, HbA1C, creatinine, lipids, albumin excretion rate (AER) as well as smoking habits was obtained from patient records. In addition, blood samples were obtained for measurement of C-peptide, GAD antibodies and for DNA. Follow-up information on metabolic control, progression of disease, development of diabetic complications is obtained from patient records. Information includes retinopathy, nephropathy, neuropathy and macrovascular diseases. Further information can be found at <https://snd.gu.se/en/catalogue/study/ext0074>.

**FinnDiane** (The Finnish Diabetic Nephropathy Study): The FinnDiane Study is a prospective nationwide multicentre study which aims to identify genetic and environmental factors affecting complications of type 1 diabetes. The volunteer participants are enrolled via their attending physicians in hospitals and healthcare centers across Finland. Further details of the study protocol have been described earlier, and additional information of the study can be found at http://www.finndiane.fi/. For this study, we selected adult patients (age > 18 years) with type 1 diabetes and age at diabetes onset < 40.

**GoKinD** (The Genetics of Kidneys in Diabetes study): The GoKinD study is an initiative that aims to identify genes that are involved in diabetic nephropathy. A large number of individuals with type 1 diabetes were screened to identify two subsets, one with clear-cut kidney disease and another with normal renal status despite long-term diabetes. Those who met additional entry criteria and consented to participate were enrolled. When possible, both parents also were enrolled to form family trios.

**EDIC** (The Epidemiology of Diabetes Interventions and Complications): The EDIC study is a multicenter, longitudinal, observational study designed to use the well-characterized The Diabetes Control and Complications Trial (DCCT) cohort of > 1,400 type 1 patients to determine the long-term effects of prior separation of glycemic levels on micro- and macrovascular outcomes. Using a standardized annual history and physical examination, 28 EDIC clinical centers that were DCCT clinics have continued to follow the EDIC cohort since 1994.

**AUST** (The Australian DR Genetics Case-Control Study): In 2007 recruitment of participants began from three tertiary hospitals in metropolitan Adelaide, South Australia: The Flinders Medical Centre, the Royal Adelaide Hospital and the Queen Elizabeth Hospital. The project was expanded to the Royal Melbourne Hospital (Melbourne, Victoria) in 2009, the Sydney Eye Hospital (Sydney, New South Wales) in 2010, and the Repatriation General Hospital (Adelaide, South Australia) and The Canberra Hospital (Canberra, Australian Capital Territory) in 2011. Ophthalmology, endocrinology and renal clinics of these hospitals were used to identify and recruit participants with diabetes meeting eligibility criteria.

**BMES** (The Blue Mountains Eye Study): The BMES is a population-based cohort study of vision and common eye diseases in a suburban population of older Australians. All noninstitutionalized residents aged 49 years or older were identified in a door-to-door census of two postcode areas in the Blue Mountains region, west of Sydney. At baseline (1992-1994) survey, 3654 (82.4%) of those eligible participated. Of these, 75.8% and 76.7% of survivors attended the 5- (1997-1999) and 10-year (2002-2004) follow-up visits, respectively. All BMES surveys were approved by the University of Sydney and Western Sydney Area Health Service Human Research Ethics Committees, and adhered to the tenets of the Declaration of Helsinki. Written informed consent was obtained from all participants at each study visit.

**CHS2** (Cardiovascular Health Study 2): The Cardiovascular Health Study (CHS) was initiated by the National Heart, Lung and Blood Institute (NHLBI) in 1987 to determine the risk factors for development and progression of cardiovascular disease (CVD) in older adults, with an emphasis on subclinical measures. The study recruited 5,888 adults aged 65 or older at entry in four U.S. communities and conducted extensive annual clinical exams between 1989-1999 along with semi-annual phone calls, events adjudication, and subsequent data analyses and publications. Further information can be obtained at <https://chs-nhlbi.org/>.

**AAPDR** (African American Proliferative Diabetic Retinopathy Study): Participants for the African American Proliferative Diabetic Retinopathy Study were recruited between 2011 and 2013 from 4 clinical sites: University of Mississippi Medical Center, Massachusetts Eye and Ear Infirmary, Boston Medical Center and Harvard Vanguard Medical Associates, USA. All participants self-identified as African American and had a known diagnosis of type 2 diabetes by 2003 American Diabetes Association criteria and/or by being on anti-diabetic medication.

**JHS** (Jackson Heart Study): The study was initiated in 1998 by the National Heart, Lung and Blood Institute (NHLBI) and the National Institute on Minority Health and Health Disparities (NIMHD) as a longitudinal investigation of genetic and environmental risk factors associated with the disproportionate burden of cardiovascular disease (CVD) in African-Americans. The JHS represents an expansion of the Jackson Field Center of the Atherosclerosis Risk in Communities (ARIC) study, to broaden data collection in an African- American population and to increase access to and participation of African American populations and scientists in biomedical research and professions. The study recruited approximately 5300 African-American men and women aged 35-84 living in the Jackson, MS, metropolitan area. It is uniquely positioned to investigate CVD risk factors, especially manifestations related to hypertension such as coronary artery disease, heart failure, stroke, peripheral arterial disease, and renal disease. Further information can be found at <https://www.jacksonheartstudy.org/>.

**ARIC** (Atherosclerosis Risk in Communities): The study is a prospective epidemiologic study conducted in four U.S. communities. ARIC is designed to investigate the etiology and natural history of atherosclerosis, the etiology of clinical atherosclerotic diseases, and variation in cardiovascular risk factors, medical care and disease by race, gender, location, and date. ARIC includes two parts: the Cohort Component and the Community Surveillance Component. The Cohort Component began in 1987, and each ARIC field center randomly selected and recruited a cohort sample of approximately 4,000 individuals aged 45-64 from a defined population in their community. The Community Surveillance Component investigates to determine the community-wide occurrence of hospitalized myocardial infarction and coronary heart disease deaths in men and women aged 35-84 years. Further information can be found at <http://www2.cscc.unc.edu/aric/>.

**MESA-AA** (Multi-Ethnic Study of Atherosclerosis-African Americans): The Multi-Ethnic Study of Atherosclerosis (MESA) is a study of the characteristics of subclinical cardiovascular disease (disease detected non-invasively before it has produced clinical signs and symptoms) and the risk factors that predict progression to clinically overt cardiovascular disease or progression of the subclinical disease. MESA researchers study a diverse, population-based sample of 6,814 asymptomatic men and women aged 45-84. Around 28 percent of the MESA population is African-American. Further information can be found at <https://www.mesa-nhlbi.org/aboutMESA.aspx>.

**The adjusted covariates used in the regression model of each cohort.**

**GoDARTS**: age, gender, HbA1c and duration of diabetes.

**SDR**: age, gender, HbA1c and duration of diabetes.

**FinnDiane**: age, gender, HbA1c, duration of diabetes, principal component 1 and 2.

**GoKinD/EDIC**: nephropathy was controlled between cases and controls before allelic association.

**AUST**: HbA1c and duration of diabetes.

**BMES, CHS2, AAPDR, JHS, ARIC, MESA-AA**: HbA1c (or fasting blood sugar), and duration of diabetes.
