# Supplementary material for: A genome‐wide association study suggests new evidence for an association of the NADPH Oxidase 4 (NOX4) gene with severe diabetic retinopathy in type 2 diabetes
Source: Acta Ophthalmol. 2018 Sep 4;96(7):e811–9. doi: 10.1111/aos.13769 (PMC6263819; doi:10.1111/aos.13769)
Supplement: Supplementary file 4 — Table S1. The clinical characteristics of the case and control populations in GoDARTS. [file AOS-96-e811-s004.docx]

**Table S1.** The clinical characteristics of the case and control populations in GoDARTS

|  | **Sex** | **Age**  **(years)** | **BMI***  **(kg/m^2^)** | **Duration of Diabetes**  **(years)** | **HbA1c**  **(%)** |
| --- | --- | --- | --- | --- | --- |
|  | **(m/f)** | **(Mean+SD)** | **(Mean+SD)** | **(Mean+SD)** | **(Mean+SD)** |
| Cases | 337/223 | 68.06+9.41 | 30.95+6.03 | 23.83+7.99 | 8.09+1.58 |
| Controls | 2288/1818 | 66.49+10.75 | 31.23+6.30 | 14.12+4.84 | 7.30+1.33 |
| P value | =0.046 | <0.001 | =0.290 | <0.001 | <0.001 |

*BMI: body mass index
