# Supplementary material for: A genome‐wide association study suggests new evidence for an association of the NADPH Oxidase 4 (NOX4) gene with severe diabetic retinopathy in type 2 diabetes
Source: Acta Ophthalmol. 2018 Sep 4;96(7):e811–9. doi: 10.1111/aos.13769 (PMC6263819; doi:10.1111/aos.13769)
Supplement: Supplementary file 5 — Table S2. Linkage disequilibrium (LD) score in the GoDARTS and in the HapMap CEU populations. [file AOS-96-e811-s005.docx]

|  | **SNP IDs** | **rs3913535** | **rs10765219** | **rs11018670** |
| --- | --- | --- | --- | --- |
| GoDARTS | rs3913535 | 1 | 0.02 | 0.02 |
|  | rs10765219 | 0.02 | 1 | 0.95 |
|  | rs11018670 | 0.02 | 0.95 | 1 |
| HapMap CEU | rs3913535 | 1 | 0.01 | 0.01 |
|  | rs10765219 | 0.01 | 1 | 0.98 |
|  | rs11018670 | 0.01 | 0.98 | 1 |

**Table S2** Linkage disequilibrium (LD) score in the GoDARTS and in the HapMap CEU populations

LD score means R-squared score.

GoDARTS: The Genetics of Diabetes Audit and Research Tayside

CEU: Caucasian
