# Supplementary material for: A genome‐wide association study suggests new evidence for an association of the NADPH Oxidase 4 (NOX4) gene with severe diabetic retinopathy in type 2 diabetes
Source: Acta Ophthalmol. 2018 Sep 4;96(7):e811–9. doi: 10.1111/aos.13769 (PMC6263819; doi:10.1111/aos.13769)
Supplement: Supplementary file 6 — Table S3. The input information and the output meta‐analysis results of 4 African American DR cohorts based on GWAMA (default setting). [file AOS-96-e811-s006.docx]

| **Cohorts** | **MARKER** | **EA** | **NEA** | **OR** | **OR_95L** | **OR_95U** | **N (cases+controls)** | ***P*** |
| --- | --- | --- | --- | --- | --- | --- | --- | --- |
| AAPDR | rs3913535 | C | T | 0.90 | 0.58 | 1.41 | 311 (255+56) | 0.65 |
|  | rs10765219 | T | G | 0.94 | 0.61 | 1.44 | 311 | 0.77 |
|  | rs11018670 | G | A | 0.79 | 0.47 | 1.31 | 311 | 0.36 |
| JHS | rs3913535 | C | T | 1.36 | 0.66 | 2.77 | 181 (21+160) | 0.40 |
|  | rs10765219 | T | G | 1.79 | 0.88 | 3.70 | 181 | 0.11 |
|  | rs11018670 | G | A | 1.58 | 0.75 | 3.33 | 181 | 0.23 |
| ARIC | rs3913535 | C | T | 0.58 | 0.21 | 1.65 | 164 (14+150) | 0.31 |
|  | rs10765219 | T | G | 0.68 | 0.28 | 1.63 | 164 | 0.38 |
|  | rs11018670 | G | A | 0.84 | 0.30 | 2.38 | 164 | 0.75 |
| MESA-AA | rs3913535 | C | T | 3.02 | 0.80 | 11.32 | 136 (6+130) | 0.10 |
|  | rs10765219 | T | G | 1.14 | 0.38 | 3.38 | 136 | 0.82 |
|  | rs11018670 | G | A | 0.32 | 0.04 | 2.42 | 136 | 0.27 |
| Meta-analysis | rs3913535 | C | T | 1.03 | 0.73 | 1.45 | 792 (296+496) | 0.88 |
| all 4 | rs10765219 | T | G | 1.04 | 0.75 | 1.44 | 792 | 0.81 |
| cohorts | rs11018670 | G | A | 0.92 | 0.63 | 1.36 | 792 | 0.69 |

**Table S3. The input information and the output meta-analysis results of 4 African American DR cohorts based on GWAMA (default setting)**

EA: effective allele

NEA: non-effective allele

OR: odds ratio

N: number

AAPDR: African American Proliferative Diabetic Retinopathy Study

JHS: Jackson Heart Study

ARIC: Atherosclerosis Risk in Communities Study

MESA-AA: Multi-Ethnic Study of Atherosclerosis-African Americans
