# Supplementary material for: A genome‐wide association study suggests new evidence for an association of the NADPH Oxidase 4 (NOX4) gene with severe diabetic retinopathy in type 2 diabetes
Source: Acta Ophthalmol. 2018 Sep 4;96(7):e811–9. doi: 10.1111/aos.13769 (PMC6263819; doi:10.1111/aos.13769)
Supplement: Supplementary file 7 — Table S4. The p values of suggestive SNPs (identified by others) in GoDARTS. [file AOS-96-e811-s007.docx]

| **Reference** | **Population** | **SNPs** | **Chr** | **Position** | **OR** | ***P*** |
| --- | --- | --- | --- | --- | --- | --- |
| Fu et al^28^ | Mexican-Americans | rs2300782 | 5 | 110788785 | 0.99 | 0.91 |
| Huang et al^29^ | Taiwanese | rs12092121 | 1 | 59153010 | 0.97 | 0.66 |
|  |  | rs13163610 | 5 | 93548877 | 0.96 | 0.71 |
|  |  | rs12219125 | 10 | 20593087 | 1.13 | 0.23 |
|  |  | rs4838605 | 10 | 49699957 | 1.01 | 0.92 |
|  |  | rs11101355 | 10 | 49723037 | 1.03 | 0.75 |
|  |  | rs11101357 | 10 | 49723300 | 1.02 | 0.79 |
|  |  | rs4462262 | 10 | 59189178 | 1.02 | 0.77 |
| Sheu et al^30^ | Taiwanese | rs9565164 | 13 | 76039376 | 1.29 | 0.11 |
|  |  | rs1399634 | 2 | 170244607 | 1.19 | 0.08 |
|  |  | rs2380261 | 13 | 96951433 | 0.97 | 0.81 |
| Grassi et al^31^ | Caucasian, USA | rs476141 | 1 | 244176424 | 0.95 | 0.52 |
| Burdon et al^32^ | Caucasian, Australia | rs9896052 | 17 | 73418862 | 1.06 | 0.53 |

**Table S4.** The *P* values of suggestive SNPs (identified by others) in GoDARTS

Some suggestive SNPs identified by others were not presented in GoDARTS.

Chr: Chromosome

OR: odds ratio
