# Supplementary material for: A genome‐wide association study suggests new evidence for an association of the NADPH Oxidase 4 (NOX4) gene with severe diabetic retinopathy in type 2 diabetes
Source: Acta Ophthalmol. 2018 Sep 4;96(7):e811–9. doi: 10.1111/aos.13769 (PMC6263819; doi:10.1111/aos.13769)
Supplement: Supplementary file 8 — Table S5. The p values of the 3 SNPs when controls (with diabetic history over 20 years) were matched with cases in terms of duration of diabetes. [file AOS-96-e811-s008.docx]

**Table S5.** The *P* values of the 3 SNPs when controls (with diabetic history over 20 years) were matched with cases in terms of duration of diabetes

| SNPs | Chr: position  （hg19） | Gene | *P* value | Odds ratio |
| --- | --- | --- | --- | --- |
| rs3913535 | 11:89096757 | *NOX4* | 8.52X10^-6^ | 1.54 |
| rs10765219 | 11:89354278 | 31kb to *NOX4* | 6.99X10^-5^ | 1.54 |
| rs11018670 | 11:89356628 | 33kb to *NOX4* | 2.84X10^-5^ | 1.53 |

Case number: 560, control number: 470

Duration of diabetes for cases: 23.83+8.0

Duration of diabetes for control: 23.86+4.4

*P*=0.9
